# QRS fragmentation does not predict mortality in survivors of acute myocardial infarction

**DOI:** 10.1002/clc.24218

**Published:** 2024-01-19

**Authors:** Julia Allescher, Daniel Sinnecker, Bernhard von Goeldel, Petra Barthel, Alexander Müller, Alexander Hapfelmeier, Eimo Martens, Karl‐Ludwig Laugwitz, Georg Schmidt, Alexander Steger

**Affiliations:** ^1^ Klinik und Poliklinik für Innere Medizin I, University Hospital Technical University of Munich Munich Germany; ^2^ DZHK (German Centre for Cardiovascular Research) Partner Site Munich Heart Alliance Munich Germany; ^3^ Medizinisches Versorgungszentrum (MVZ) Harz Goslar Germany; ^4^ School of Medicine, Institute of AI and Informatics in Medicine Technical University of Munich Munich Germany; ^5^ School of Medicine, Institute of General Practice and Health Services Research Technical University of Munich Munich Germany

**Keywords:** acute myocardial infarction, fragmented QRS complex, electrocardiogram, mortality, noninvasive risk stratification

## Abstract

**Background:**

Despite advances in coronary revascularization and in heart failure management, myocardial infarction survivors remain at substantially increased mortality risk. Precise risk assessment and risk‐adapted follow‐up care are crucial to improve their outcomes. Recently, the fragmented QRS complex, i.e. the presence of additional spikes within the QRS complexes on a 12‐lead electrocardiogram, has been discussed as a potential non‐invasive risk predictor in cardiac patients.

**Hypothesis:**

The aim of this study was to evaluate the prognostic meaning of the fragmented QRS complex in myocardial infarction survivors.

**Methods:**

609 patients with narrow QRS complexes <120 ms were included in a prospective cohort study while hospitalized for myocardial infarction and followed for 5 years.

**Results:**

The prevalence of the fragmented QRS complex in these patients amounted to 46.8% (285 patients). These patients had no increased hazard of all‐cause death (HR 0.84, 95%–CI 0.45–1.57, *p* = 0.582) with a mortality rate of 6.0% compared to 7.1% in patients without QRS fragmentations. Furthermore, the risks of cardiac death (HR 1.28, 95%–CI 0.49–3.31, *p* = 0.613) and of non‐cardiac death (HR 0.6, 95%–CI 0.26–1.43, *p* = 0.25) were not significantly different in patients with QRS fragmentations. However, patients with QRS fragmentations had increased serum creatine kinase concentrations (1438U/l vs. 1160U/l, *p* = 0.039) and reduced left ventricular ejection fractions (52% vs. 54%, *p* = 0.011).

**Conclusions:**

The hypothesis that QRS fragmentation might be a prognostic parameter in survivors of myocardial infarction was not confirmed. But those with QRS fragmentation had larger myocardial infarctions, as measured by creatine kinase and left ventricular ejection fraction.

## Graphical abstract

The aim of this study was to assess whether fragmented QRS (fQRS) was a mortality predictor in acute myocardial infarction survivors. Six hundred nine patients were included and followed for 5 years. fQRS prevalence was 46.8%. These patients had no increased hazard of all‐cause death (hazard ratio: 0.84, 95% confidence interval: 0.45–1.57, *p* = .582) with a mortality rate of 6.0% compared to 7.1% in those without fQRS.

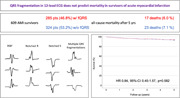

**Abbreviations**
95% CI: 95% confidence interval
ACE‐inhibitor: angiotensin‐converting enzyme inhibitor
ACM: all‐cause mortality
AMI: acute myocardial infarction
ART: Autonomic Regulation Trial
BMI: body mass index
CABG: coronary artery bypass grafting
CM: cardiac mortality
COPD: chronic obstructive pulmonary disease
ECG: electrocardiogram
eGFR: estimated glomerular filtration rate
fQRS: fragmented QRS
GRACE score: Global Registry of Acute Coronary Events score
HR: hazard ratio
IQR: interquartile range
LVEF: left ventricular ejection fraction
PCI: percutaneous coronary intervention

## 1 INTRODUCTION

In recent years, improvements in percutaneous coronary revascularization and modern heart failure management have reduced mortality, morbidity, and rehospitalization rates in patients who suffer an acute myocardial infarction (MI).^1^, ^2^, ^3^, ^4^, ^5^, ^6^ Nevertheless, survivors of MI remain at substantially increased mortality risk compared to the general population.^2^, ^7^ Therefore, precise risk stratification and risk‐adapted personalized follow‐up care are of utmost importance in these individuals aiming for enhanced overall prognosis and quality of life and alleviation of the disease burden on the healthcare system. In current guidelines,^8^, ^9^ a reduced left ventricular ejection fraction is considered one of the major risk predictors in survivors of acute MI. However, patients with a preserved left ventricular ejection fraction also frequently suffer from cardiac arrhythmias and cardiac death on the one hand, while on the other hand, many patients with severely reduced left ventricular ejection fraction never experience any severe cardiovascular event.^10^, ^11^ Thus, additional, easily accessible, low‐cost, and noninvasive stratification tools are urgently needed to better identify patients at risk.

Recently, fragmentations of the QRS complex (fQRS) in resting 12‐lead electrocardiograms (ECG) have been discussed as a potential simple risk predictor in cardiac patients.^12^, ^13^, ^14^, ^15^, ^16^, ^17^, ^18^ Narrow QRS complexes with an unremarkable morphology are found in patients with a normal electrical excitation propagation in the ventricles. If the electrical excitation propagation is impaired, for example, due to areas of myocardial fibrosis or scar,^13^, ^19^ cardiomyopathies,^20^ channelopathies,^21^, ^22^ or systemic diseases with cardiac involvement such as sarcoidosis,^23^ QRS morphology can be altered or fragmented. Associations between the presence of fragmented QRS complexes and cardiac death, all‐cause mortality, and cardiac complications have been described.^12^, ^14^, ^15^, ^16^, ^17^, ^19^, ^24^, ^25^, ^26^ Nevertheless, fragmented QRS complexes are also present in approximately 5% of healthy adults.^12^, ^27^, ^28^ So far, the evidence about the prognostic relevance of the fragmented QRS complex in different clinical circumstances is limited and not very clear. The aim of this study was to narrow this evidence gap for a subset of MI patients.

## 2 METHODS

### 2.1 Study population and follow‐up

The Autonomic Regulation Trial (ART, NCT00196274 ClinicalTrials.gov) is a prospective cohort study conducted at the University Hospital of the Technical University of Munich, Germany, and at the German Heart Center Munich, Germany. Between May 2000 and March 2005, survivors of acute MI (ST‐elevation MI and non‐ST‐elevation MI) were included in this study during the initial hospitalization due to the index MI. Additional inclusion criteria were age ≤80 years, sinus rhythm, no secondary prophylaxis indication for an implantable cardioverter defibrillator. Patients with bundle branch block ECG patterns (QRS ≥ 120 ms) were excluded from the here presented substudy. The study was approved by the ethics committee of the Technical University of Munich and informed consent was obtained from all participants. The study was conducted in accordance with the current guidelines and with the Declaration of Helsinki.

The participating patients were followed for a median of 5.0 years. Patients who did not attend scheduled follow‐up visits were contacted via mail, telephone, or their general practitioner. The last option was to contract the residents' registration office. The last follow‐up was performed in May 2010. The primary endpoint was all‐cause mortality.

### 2.2 Clinical data

To establish the diagnosis of an acute MI, at least two of the following criteria were required: typical chest pain, creatine kinase above twice the upper limit of normal, and admission ST segment elevations that were diagnostic for MI. The left ventricular ejection fraction assessment was performed either by echocardiography (biplane Simpson's method) or by left ventricular angiography. The Global Registry of Acute Coronary Events score is a recognized clinical risk score that characterizes patients who present with acute coronary syndrome. It combines age, serum creatinine, previous history of MI, congestive heart failure, in‐hospital percutaneous coronary intervention, resting heart rate, systolic blood pressure, ST segment deviation, and positive cardiac enzymes. The score ranges from 1 (lowest risk) to 210 points (highest risk).^29^ Diabetes mellitus was considered present if a patient was already diagnosed and was receiving treatment or if occasional plasma glucose levels repeatedly exceeded 11 mmol/L.

### 2.3 ECG analysis

Resting 12‐lead ECGs were recorded during the index hospitalization at a standard printing speed of 50 mm/s and an amplitude of 10 mm/mV. The ECG analysis including the detection of fragmented QRS complexes was performed independently by two experienced physicians, who were blinded to all follow‐up data. The results of both interpreters were then compared. There was 99% concordance for the detection of fragmented QRS complexes between both readers. In case of disagreement, a third independent interpreter was consulted, and a mutual consent was found.

**Figure 1:**
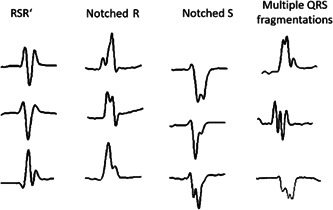
Different QRS fragmentation morphologies.

The fragmented QRS pattern in narrow QRS complexes (<120 ms) comprises different morphologies including the presence of one or multiple additional R waves (R′) or notches in two contiguous leads corresponding to a major coronary artery territory (anterior, lateral, and inferior wall)^19^, ^26^ (Figure 1).

### 2.4 Statistical analysis

The distribution of continuous variables is presented as medians and interquartile ranges (IQRs). Categorical variables are presented as absolute and relative frequencies. Hypothesis tests for group differences of continuous variables were performed by Mann–Whitney *U* tests. Hypothesis tests for group differences of categorical variables were performed by *χ* ^2^ tests. Median follow‐up was estimated by the reverse Kaplan–Meier method. Univariable Cox proportional hazard models were used for effect estimation by hazard ratios (HRs) and hypothesis testing by *z* tests for the association of variables and right‐censored outcome measures. Precision of estimates was described by 95% confidence intervals (95% CI). All hypothesis tests were two‐sided and performed at 5% significance levels. Survival curves were generated by the Kaplan–Meier method. Analyses were performed in IBM SPSS Statistics, version 28 (IBM Corp) and in R 3.6.1 (The R Foundation for Statistical Computing).

## 3 RESULTS

### 3.1 ECG analysis

The baseline characteristics of the 609 survivors of acute MI are presented in Table 1. Resting 12‐lead ECGs were obtained a median of 4 days (IQR: 2–7 days) after the index MI. QRS fragmentation was present in 285 patients (46.8%). In 206 patients (72.3%) QRS fragmentation was present in leads corresponding to the inferior segments, in 115 patients (40.4%) QRS fragmentation was present in leads corresponding to the anterior myocardial segments, and in 42 patients (14.7%) QRS fragmentation was present in leads corresponding to the lateral segments. Repeatedly, fragmented QRS complexes were seen in more than one major coronary artery territory. Most commonly, they were simultaneously found in anterior and inferior leads (*n* = 40, 14.0%). In 11 patients (3.9%) QRS fragmentations were simultaneously found in inferior and lateral leads as well as in anterior and lateral leads, respectively. QRS fragmentations in all coronary artery territories were present in eight patients (2.8%).

**Table 1:** Clinical characteristics of the study population.

	All	fQRS	No fQRS	*p*
Number of patients, *n* (%)	609 (100)	285 (46.8)	324 (53.2)	
Age (years), median (IQR)	60.9 (51.8–68.6)	60.2 (51.5–67.9)	61.4 (51.9–69.1)	.327
Female, *n* (%)	119 (19.5)	45 (15.8)	74 (22.8)	.029
Diabetes mellitus, *n* (%)	120 (19.7)	58 (20.4)	62 (19.1)	.707
GRACE score, median (IQR)	110 (93–126)	109 (91.5–126)	111 (95–126)	.614
eGFR_MDRD_ (mL/min), median (IQR)	74.1 (63.1–86.9)	72.8 (62.3–85.2)	74.6 (63.5–88.3)	.22
LVEF (%), median (IQR)	52 (45–60)	52 (43–59)	54 (46–61)	.011
COPD, *n* (%)	20 (3.3)	5 (1.8)	15 (4.6)	.047
History of AMI, *n* (%)	50 (8.2)	27 (9.5)	23 (7.1)	.287
Creatinine kinase (U/L), median (IQR)	1302 (636–2460)	1438 (666–2640)	1160.0 (568.3–2250.0)	.039
BMI (kg/m²), median (IQR)	26.7 (24.5–29.3)	26.7 (24.7–28.7)	26.7 (24.5–29.7)	.653
Intervention				
PCI, *n* (%)	578 (94.9)	274 (96.1)	304 (93.8)	.195
Thrombolysis, *n* (%)	2 (0.3)	1 (0.4)	1 (0.3)	.928
CABG, *n* (%)	2 (0.3)	1 (0.4)	1 (0.3)	.928
Medication				
Aspirin, *n* (%)	585 (96.1)	271 (95.1)	314 (96.9)	.248
ACE‐inhibitor, *n* (%)	569 (93.4)	263 (92.3)	306 (94.4)	.282
Beta‐blockers, *n* (%)	577 (94.7)	270 (94.7)	307 (94.8)	.993
Statins, *n* (%)	571 (93.8)	267 (93.7)	304 (93.8)	.942
Diuretics, *n* (%)	273 (44.8)	133 (46.7)	140 (43.2)	.392

Abbreviations: ACE‐inhibitor, angiotensin‐converting enzyme inhibitor; AMI, acute myocardial infarction; BMI, body mass index; CABG, coronary artery bypass grafting; COPD, chronic obstructive pulmonary disease; eGFR, estimated glomerular filtration rate; fQRS, fragmented QRS; GRACE score, global registry of acute coronary events score; IQR, interquartile range; LVEF, left ventricular ejection fraction; PCI, percutaneous coronary intervention.

**Figure 2:**
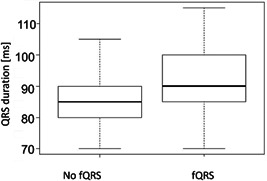
QRS duration in patients with and without fragmented QRS complexes. Boxplots show medians and interquartile ranges.

The median QRS duration in the study population was 90 ms (IQR: 80–95 ms). QRS duration was longer in patients with fragmented QRS complexes (90 ms, IQR: 85–100 ms) compared to patients with normal QRS morphology (85, IQR: 80–90 ms). This difference was statistically significant (*p* < .001, Figure 2).

### 3.2 Patient characteristics in groups with and without fragmented QRS complexes

Baseline characteristics in patients with and in patients without fragmented QRS complexes are shown in Table 1. Compared to the patients without QRS fragmentations, the patients with QRS fragmentations had more severe MIs as quantified by higher serum creatine kinase concentrations (1438 vs. 1160 U/L, *p* = .039) and a lower left ventricular ejection fraction (52% vs. 54%, *p* = .011). Interestingly, the proportion of women was higher in patients without fragmented QRS (22.8%) compared to patients with fragmented QRS (15.8%, *p* = .029), meaning that in the here presented cohort of MI survivors fragmented QRS was more frequent in men (49.0%) than in women (37.8%). Medical and interventional therapy was not different in both groups.

### 3.3 Outcome data

After 5.0 years of follow‐up, 40 patients (6.6%) had died. 17 patients (2.8%) suffered a cardiac death, 23 patients (3.8%) suffered a noncardiac death, and five patients (0.8%) suffered a sudden cardiac death. In the subgroup of patients with fragmented QRS complexes 17 patients died (6%) and in the subgroup of patients without fragmented QRS complexes 23 patients died (7.1%). Thus, a fragmentation of the QRS complex was not associated with a higher risk of all‐cause death in the here presented subset of MI survivors. This finding was confirmed by a Cox proportional hazard model yielding an HR of 0.84 (95% CI: 0.45–1.57, *p* = .582). Consistently, the fragmented QRS was neither a significant predictor of cardiac mortality (HR: 1.28, 95% CI: 0.49–3.31, *p* = .613) nor of noncardiac mortality (HR: 0.6, 95% CI: 0.26–1.43, *p* = .25) (Table 2). Concordant results were achieved, if patients with incomplete right bundle branch block were excluded from the analyses (data not shown).

**Table 2:** Cox regression model: Estimated risk depending on presence of QRS fragmentations.

	All (*n* = 609)	fQRS (*n* = 285)	No fQRS (*n* = 324)	HR	95% CI	*p*
ACM	40 (6.6%)	17 (6%)	23 (7.1%)	0.84	0.45–1.57	.582
CM	17 (2.8%)	9 (3.2%)	8 (2.5%)	1.28	0.49–3.31	.613
Non‐CM	23 (3.8%)	8 (2.8%)	15 (4.6%)	0.6	0.26–1.43	.25

Abbreviations: 95% CI, 95% confidence interval; ACM, all‐cause mortality; CM, cardiac mortality; fQRS; fragmented QRS; HR, hazard ratio.

**Figure 3:**
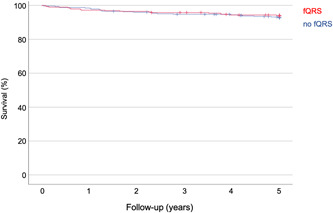
Kaplan–Meier analysis indicating no difference between patients with and patients without fragmented QRS complexes.

Figure 3 illustrates the corresponding Kaplan–Meier survival curves showing no difference between both groups of patients.

We also investigated whether the localization of QRS fragmentations (i.e., anterior, inferior, or lateral leads) had any prognostic significance. These analyses revealed that neither fragmented QRS complexes in anterior leads (HR: 0.76, 95% CI: 0.32–1.81, *p* = .535) nor in lateral leads (HR: 1.52, 95% CI: 0.54–4.26, *p* = .429) nor in inferior leads (HR: 0.84, 95% CI: 0.43–1.65, *p* = .606) were significantly associated with an increased all‐cause mortality risk. Furthermore, all‐cause mortality did not correlate with the number of ECG‐leads with overt fragmented QRS complexes (HR for each additional ECG‐lead 0.98, 95% CI: 0.82–1.16, *p* = .767).

Despite these data rejecting the fragmented QRS as a prognostic parameter in survivors of an acute MI, a trend could be observed showing that QRS fragmentations were more frequent in patients who died of a cardiac cause (52.9%) and in patients who died of sudden cardiac death (60%) compared to patients who died of a noncardiac cause (34.8%) or of any cause (42.5%).

## 4 DISCUSSION

The ART study is a prospective cohort study in which patients were enrolled after surviving an acute MI.^30^ The fQRS substudy investigated whether the fragmented QRS complex could be used as a prognostic parameter in this patient population. Patients participating in the study had a 12‐lead ECG obtained after enrollment. They were followed for 5 years.

Recently, some authors have reported that the prevalence of the fragmented QRS complex might be higher in survivors of an acute MI compared to the general population.^15^, ^31^ QRS fragmentation might also be an indicator of myocardial scar or fibrosis^13^, ^19^, ^25^, ^26^ and associated with a poor prognosis.^25^, ^26^, ^32^, ^33^, ^34^ However, other authors have not been able to confirm these observations.^31^, ^35^, ^36^

In the ART study, 285 out of 609 post‐MI patients (46.8%) had QRS fragmentations on the resting 12‐lead ECG. This prevalence is considerably higher than in the general population—where it is just over 5%.^12^, ^27^, ^28^ The presence of QRS fragmentation was associated with larger MIs as measured by total serum creatine kinase levels (1438.0 vs. 1160.0 U/L, *p* = .039) and with slightly lower left ventricular ejection fraction (52% vs. 54%, *p* = .011). In addition, the QRS complexes of patients with fragmentation were significantly wider than those of patients without QRS fragmentation (90 vs. 85 ms, *p* < .001). These results indirectly support the hypothesis that QRS fragmentation may be associated with myocardial damage (scarring or fibrosis) after MI and may be a sign of pathologically altered conduction in the myocardium.^19^, ^26^ It is also worth mentioning that the percentage of women was significantly higher in the group without a fragmented QRS complex (22.8%) than in the group with a fragmented QRS complex (15.8%). Thus, in this study, a fragmented QRS complex was more frequent in men than in women. Consequently, sex‐specific aspects should be specifically investigated in future studies. Otherwise, the distribution of patient characteristics was homogeneous in both groups. There were no differences in cardiovascular risk profile, GRACE risk score, medical or interventional therapy.

Regarding the primary endpoint, the results of this study do not support the hypothesis that the fragmented QRS complex was a prognostic parameter for all‐cause mortality in survivors of acute MI. Neither all‐cause mortality nor cardiac or noncardiac mortality occurred significantly more often in patients with a fragmented QRS complex than in those without. There were numerical trends in the prevalence of fQRS toward cardiac death and sudden cardiac death compared to noncardiac death and all‐cause death, though. Furthermore, the risk of death did not significantly depend on the number of ECG leads with fragmented QRS complexes or the location of the fragmented QRS complexes in a 12‐lead ECG (anterior, posterior, lateral leads). Interestingly, fragmented QRS complexes were most often found in inferior leads.

The study is subject to several limitations, including the constraint of being conducted within the time frame of 2000 to 2010. Another limitation is the event rate in some secondary analyses. With 17 cardiac and 23 noncardiac deaths, the event rate in these subgroups was relatively low, and possible small effects could therefore be masked. Due to the very rare occurrence of sudden cardiac death in the studied cohort (*n* = 5), no conclusion can be made about sudden cardiac death as an endpoint. The absence of follow‐up ECGs can also be considered a limitation in the ART study. Therefore, no statement can be made regarding the persistence of the observed QRS fragmentations. Indeed, some studies have reported that QRS fragmentation is of prognostic significance only if it persists over a longer period of time.^31^, ^37^ Furthermore, visual assessment of QRS morphology in the search for fragmented QRS complexes is currently still based on the partly subjective judgment of the respective examiner. Despite clearly defined morphological criteria, this offers room for misinterpretation in cases of occasionally inconclusive findings (e.g., in cases of poor ECG quality). This potential source of bias was to be avoided in the present study by having two examiners independently analyzing all 12‐lead ECGs and subsequently comparing their results. Standard ECG filter settings might mask high‐frequency fragmentations in the QRS complex.^38^ A recently published study has shown that microfragmentations beyond the visible resolution of a standard ECG can be used to determine mortality.^39^ Of course, the results of this study only apply to a selected population of postinfarction patients. No conclusions should be drawn regarding patient groups that were not examined as part of the study, such as those aged over 80 years or even the general population. Morphological definitions of QRS fragmentation in patients with wide QRS complex (≥120 ms) are different from patients with narrow QRS complex.^40^, ^41^ As patients with a wide QRS complex were excluded from this study, no statement can be made about the fragmented wide QRS complex. It should also be mentioned that the prevalence of the fragmented QRS complex in the general population is at least 5%.^27^ Furthermore, it has been described^28^ that people with a fragmented QRS complex but without overt heart disease do not have an increased risk of death.^28^ From this point of view, the fragmented QRS complex may have been present in some patients in the ART study before the index MI without a pathological correlate or due to a pre‐existing heart disease or systemic disease with cardiac involvement. This cannot be verified based on the present study data. Presumably, a more detailed classification of the different morphologies of the fragmented QRS complex might be helpful to distinguish high‐risk morphologies from harmless morphologies.^40^

## 5 CONCLUSIONS

In conclusion, the present study provides evidence that the fragmented QRS complex may indeed be an expression of myocardial damage caused by MI. However, the primary hypothesis that it may be a prognostic parameter in survivors of MI could not be confirmed.

## CONFLICT OF INTEREST STATEMENT

The authors declare no conflict of interest.

## Data Availability

The data that support the findings of this study are available from the corresponding author upon reasonable request.
